# The impact of group activities and their content on persons with dementia attending them

**DOI:** 10.1186/s13195-018-0357-z

**Published:** 2018-04-05

**Authors:** Jiska Cohen-Mansfield

**Affiliations:** 1 Minerva Center for the Interdisciplinary Study of End of Life, Tel-Aviv University, P.O.B. 39040, Ramat Aviv, 6139001 Tel-Aviv, Israel; 2 Department of Health Promotion, School of Public Health, Sackler Faculty of Medicine, Tel-Aviv University, P.O.B. 39040, Ramat Aviv, 6139001 Tel-Aviv, Israel; 3 The Herczeg Institute on Aging, Tel-Aviv University, P.O.B. 39040, Ramat Aviv, 6139001 Tel-Aviv, Israel

**Keywords:** Dementia, Group activities, Mood, Cognitive impairment

## Abstract

**Background:**

Individuals suffering from dementia and residing in nursing homes often feel lonely and bored. This study examined the engagement and mood of people with dementia in group activities, and how personal characteristics, such as cognitive function, may impact on an individual’s responses to group activities.

**Methods:**

The study included 102 participants, who took part in group activities while their mood and engagement levels were observed. Participants were invited to attend 10 different types of group activities, each of which was offered twice.

**Results:**

Results found improved engagement and mood during group activities as compared to control no-group times. Significant relationships between the type of activity and ratings of engagement and mood were also found. Although participants with higher levels of cognitive functioning manifested greater responsiveness to groups, the pattern of response to different contents did not differ by cognitive function.

**Conclusions:**

This study shows the potential utility of group activities for improving quality of life of persons with dementia and demonstrates a methodology that can be used for quality improvement to optimize group contents. Future research should expand the range of contents of group activities in order to enhance the options for improving mood and engagement of individuals with dementia.

## Background

Persons with dementia residing in nursing homes and long-term care settings are often not engaged in any activity [1]. Low levels of engagement and understimulation are problematic because they can result in boredom and loneliness that may lead to behavior challenges [1]. A potential way to mitigate these issues is to engage multiple persons with dementia in group activities. Group activities can provide individuals with dementia the opportunity to interact with both staff members and other residents within a social context while being engaged in an activity. Therefore, it is important to understand the impact of group activities on engagement and the possible related outcomes. The Comprehensive Process Model of Group Engagement serves as a conceptual framework to understand engagement of persons with dementia within group activity settings [2]. This model posits that environmental (e.g., physical and group environments), stimulus (e.g., content of activities), and personal attributes (e.g., age, cognitive functioning) impact on the engagement and affect levels of individuals taking part in the activity, which can, in turn, influence behaviors of persons with dementia. In particular, stimulus attributes, such as the content of a specific group activity, may differentially impact engagement and may also interact with other attributes, such as characteristics of group members.

Based on this model, it is possible that certain activities may be more engaging than others, depending on the characteristics of the activities. Some insight comes from a small descriptive study of 23 individuals who were diagnosed with dementia [3] that compared the engagement level in five different types of group activities—music, art, exercise, cognitive, and functional household activities. Active participation levels were highest for the exercise, music, and art groups. Although this study provides some insight into certain activities that may be more engaging, in that exercise, music, and art elicited higher active participation levels than the cognitive and household activities, no statistical tests comparing the activities were reported. The small sample size of higher functioning persons with dementia (who were able to give their own consent to the study) and the limited range of activities also make it challenging to generalize these results to other settings.

A larger study with 107 participants [4] found that activity interventions—some of which were completed in groups—engaged residents 84% of the time overall, with an average engagement duration of 27 min. Examples of activities that resulted in engagement 98–99% of the time included drama, wine/cheese social, garden, and dancing activities. These results are encouraging because they demonstrate that persons with dementia are capable of showing high engagement levels in different types of activities. However, this study tailored activities to each individual, which may have contributed to high engagement levels. Individualization of activities may not always be feasible in resource-limited facilities, so there remains a need for research that compares common group activities (e.g., exercise, reading) that have not been individualized.

Other studies have examined different outcomes that may be related to engagement, such as wellbeing and affect. For example, one study compared levels of wellbeing of persons with dementia during reminiscence group therapy, general activities (e.g., crafts and games), and unstructured time [5]. Wellbeing was observed to be lower during unstructured time compared to the activities. Wellbeing was also higher for reminiscence therapy than for general group activities. The superiority of activity over unstructured time was also demonstrated by Phillips et al. [6], who found significantly more pleasure during a group storytelling than during an unstructured control condition.

Other studies have also compared two or three group activities on outcomes other than engagement, but have not found significant differences in affect between music and reading activities [7], or in behavior when comparing a music activity versus general recreational activities, such as puzzle games and cooking [8], or else they reported inconclusive or unclear results [9]. The limited evidence, together with different study methodologies, makes it difficult to conclude which group activities may have the most promising impact, and how they compare to unstructured time. The sample sizes of four of these studies were small (fewer than 50 participants [3, 5, 7, 9]), with some consisting of relatively higher functioning persons with dementia who were capable of making their own responses on questionnaires (e.g., [7]). Such limitations make it challenging to provide recommendations for group activities that would be generalizable to a range of persons with dementia. Thus, there is a need to clarify the impact of group activities in general (i.e., in comparison to unstructured time) and to elucidate the impact of different activities on various outcomes.

To address this research gap, this study measured the engagement and mood of persons with dementia with various levels of functioning during 10 group activities. The first aim was to compare the engagement level of participants during group activities to a control condition consisting of unstructured time. The hypothesis was that group activities would be more engaging than unstructured time. The second aim was to assess whether certain activities were more engaging than others. The general hypothesis was that certain group activities would be more engaging than others, but the lack of clear findings in the literature did not lend itself to specific predictions as to which these activities would be. The third aim was to examine whether cognitive level, a personal characteristic of participants, changes the impact of the different group activities.

## Methods

### Participants

Persons with dementia were recruited from nine units at a geriatric residential facility consisting of a nursing home, a community adult day program, and an independent living facility. Inclusion criteria included: a diagnosis of dementia (derived from the medical chart) and informed consent. The criteria for exclusion were: a diagnosis of bipolar disorder; a lifelong diagnosis of schizophrenia; Cognitive Performance Scale (CPS) [10] score of 1 or lower (higher scores indicate lower function), because it is believed that people with higher levels of cognitive function can articulate their interests and needs most of the time; no dexterity movement in either hand; cannot be comfortably seated in a chair or wheelchair, or cannot be moved to the location of the group therapeutic recreation; and never spoke any English. This information was gathered from computerized charts using Minimum Data Set (MDS) version 2.0 [11, 12]. We purposefully did not exclude potential participants on the level of cognitive function, as our aim was to examine the feasibility and outcomes of group activities for persons with all stages of dementia.

### Ethics, consent, and permissions

This study was approved by the Research Ethics Board (REB) of Baycrest Health Sciences. We contacted the persons responsible for making decisions for those meeting inclusion criteria. Of the 217 contacted, 22 refused and informed consent was obtained for 105 study participants.

### Procedure

Once consent was obtained for 10–12 eligible participants for each unit, recruitment was stopped, on the assumption that 10–12 participants would be an appropriate size for a group. One participant who enrolled in the study was subsequently excluded due to the onset of illness prior to the first session. The analyses reported in this article were conducted with a final sample size of 104 study participants. Participants were grouped into nine units, with each unit having between 10 and 13 participants for whom consent was obtained. Occasionally, group activities were joined by clients who were not enrolled in the study and dropped in on the groups. The total group size ranged from 3 to 17 for the group activity sessions, with a mean of 9.2 (SD = 3.1). In total, 102 participants with consent actually attended groups, and the following text pertains to them.

Baseline characteristics were collected from the MDS and medical charts: age, sex, language, education, marital status, number of children, mobility, vision, hearing, speech, number of medications, number of diagnoses, cognitive functioning (via the CPS), and activities of daily living (via the Activities of Daily Living Long form; ADL-Long Form, obtained through the MDS [13]). Over half of the persons with dementia were female (63.7%), and the average age was 87.20 years (SD = 8.44, ranging from 59 to 101 years). Cognitive functioning assessed via the CPS [10] averaged 3.09 (SD = 1.31; range 2–6; scale, 0 = ‘Intact’ to 6 = ‘Very Severe’). ADL-Long Form [13] performance averaged 16.9 (SD = 18.98, range 0–28; scale, 0 = ‘independent’ to 28 = ‘total dependence’).

Participants were invited to participate in two sessions of each of 10 group activities which had been selected based on therapeutic recreation (TR) staff expertise and are considered to be common activities in nursing homes and long-term care settings: exercise; active physical games, such as bowling or ring toss; reminiscence poetry; baking; choral singing; creative storytelling; reading aloud with discussion; brain games/fitness; holiday discussion; and holiday newsletter activities (for additional details see [14]). One group activity was conducted per session, and each group activity was conducted twice, in random order, so that each unit completed 20 sessions of group activities. Over nine units, this resulted in a total of 180 sessions. Each unit had one TR who led all the group activities for that unit, with the exception of one unit in which the group activities were shared amongst two TRs. Each session lasted approximately 30 min, and all were conducted in English. The study took place for approximately 3–4 months on each unit and lasted 7 months overall.

The control condition consisted of observations of study participants during one or two visits in their residential location during unstructured time (i.e., when organized activities were not being conducted). These 3–5-min observations were scheduled to take place at a random order with respect to the other group sessions.

Observations of participants during group activities were independently made by a trained research assistant, trained student volunteers, and the TR who had led the group activity (after its completion). The control condition sessions were observed by at least one TR or research staff observer (often both).

#### Assessments

##### Outcome measures

Observations were collected using the Group Observational Measurement of Engagement (GOME) [2] and included outcomes of engagement, mood, and sleep. These are described in the following.

The construct of **engagement** was measured by three variables: ***‘engagement’,*** ‘*How much of the group was the participant engaged in the group activity*?’, rated on a scale from 0 = none of the time to 5 = most or all of the time; ***active participation,*** ‘*To what extent did the participant actively participate in the group?’,* rated from 0 = not at all to 4 = very much*; and* ***attitude towards the activity*** *(‘most of the time’),* ranging from 1 = very negative to 7 = very positive.

Active participation and attitude were not measured in the control observations, since most often there was no particular stimulus or activity to actively participate with or to have an attitude toward.

Because the term ‘engagement’ is used to describe a group of variables as well as a specific indicator variable, we will use quotes whenever referring to the particular observed variable named ‘engagement’ and use the term without quotes when we refer to the general construct of engagement.

***Positive mood*** was measured through observation, with ratings ranging from 0 = not at all to 4 = very much.

***Sleep*** and sleepiness was rated on the basis of observation on a scale ranging from 0 = none of the time to 6 = all of the time.

More detailed descriptions of the variables representing engagement, mood, and sleep, together with their psychometric properties showing good inter-rater reliability and validity, are presented in [2, 15].

### Analytical approach

In order to compare ratings during groups to ratings for control observations, we aggregated all group observations across the 20 group experiences of each of the participants and also aggregated the two control observations for each participant. Paired *t* tests were used to compare engagement, mood, and sleep for each participant during group observations vs control observations. Effect size for those paired *t* tests were calculated as: (mean_diff_ / SD_diff_) × √2_._

To address the second goal, of assessing the relative impact of the different group activities on participants, we used a mixed model analysis in order to address the repeated measures in the data, which included two observations per participant per group topic done by two different observers. The mixed model analysis also enabled us to address the missing data resulting from uneven participation in the groups, which we will examine in another paper. In order to reduce the number of models we examined, we used the average of observations by TR and by the other observer as the dependent variable. In the mixed model, we included cognitive function as a covariate, because we have found it to be the most important personal variable affecting engagement and mood in this population [16]. Holiday discussion was used as the reference group to which other groups were compared.

In order to examine the third goal, determining whether the relative impact of different group topics varied by cognitive function, we divided the population into two groups, those of moderate cognitive impairment (CPS rating of 2 and 3; *n* = 55) vs advanced cognitive impairment (CPS ratings of 4, 5, and 6; *n* = 47). We then conducted the same mixed model analysis described for the second goal within each subgroup and examined these results for the two subgroups for ‘engagement’, active participation, and positive mood. Due to the small group sizes, we examined effects with *p* < 0.10 in this section.

## Results

### Group activities vs control condition

Table 1 presents the comparison of the group observations to the control observations, showing that during group activities participants were more engaged, displayed a more positive mood, and spent less time asleep or displaying sleepiness than during the control observations. This was found regardless of whether TR staff or research observations were used, although effect sizes were larger when using TR ratings.

**Table 1:** Comparison of mean engagement, mood, and sleep levels during group activities vs during control observations

Variable	Rater	Group activity		Control		*t*	df	*p* (two-tailed)	Effect size
		Mean	SD	Mean	SD				
'Engagement'	TR	3.55	1.41	2.72	1.96	4.84	92	0.000	0.71
	Observer	3.73	1.35	3.31	1.7	3.43	96	0.001	0.49
Positive mood	TR	2.48	1.07	1.76	1.28	6.04	91	0.000	0.89
	Observer	1.52	1.07	1.24	1.29	3.12	96	0.002	0.45
Asleep	TR	1.14	1.62	1.87	2.15	−3.87	89	0.000	−0.58
	Observer	1.19	1.63	1.48	2.09	−1.94	94	0.056	−0.28

*SD* standard deviation, *df* degrees of freedom, *TR* therapeutic recreation

### Comparison of group activities

**Fig. 1:**
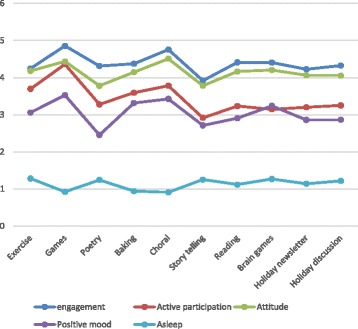
Mean impact on different outcomes, after controlling for cognitive function. Note: different outcome variables are not comparable, as they involve different scales. *Engagement scale,* 0 = none of the time to 5 = most or all of the time; *active participation,* 0 = not at all to 4 = very much; *attitude,* 1 = very negative to 7 = very positive; *positive mood,* 0 = not at all to 4 = very much; *sleep scale,* 0 = none of the time to 6 = all of the time

The mixed model analysis found statistically significant effects for group topic and for cognitive function in each of the analyses, with *p* < 0.001 for ' engagement', active participation, attitude, and positive mood, and *p* = 0.018 in the comparison of the different group activities on sleep and sleepiness (Table 2). The much higher values for *F* statistics associated with cognitive functioning as compared to group activities suggest that cognitive function had a greater impact on the outcome variables than did the differences among group topics. When comparing the different activities to the reference group of holiday discussion, games and choral groups resulted in significantly more ‘engagement’, active participation, positive attitude, and positive mood, and also in significantly less sleepiness; exercise groups resulted in significantly more active participation; brain games resulted in significantly more positive mood; and baking was associated with significantly more active participation and positive mood; in contrast, poetry resulted in a worse attitude and less positive mood, and storytelling resulted in significantly less ‘engagement’, less active participation, and a less positive attitude (Table 3). The estimated means of the dependent variables for the different groups (after controlling for cognitive function) are displayed in Fig. 1. The figure shows that the different dependent variables tended to fluctuate in a similar manner for the different activities, with sleep naturally showing the mirror image of the fluctuations and also manifesting smaller changes among activities.

**Table 2:** Mixed model analyses comparing the impact of different group activities while controlling for cognitive function

Variable	Group topic effect	CPS effect
'Engagement'	*F*_9, 1097_ = 7.03***	*F*_1,100_ = 87.35***
Active participation	*F*_9, 1097_ = 14.55***	*F*_1, 102_ = 87.72***
Attitude	*F*_9, 1108_ = 11.50***	*F*_1, 104_ = 63.07***
Positive mood	*F*_9, 1100_ = 9.16***	*F*_1,99_ = 66.26***
Sleep	*F*_9,1030_ = 2.24*	*F*_1,105_ = 48.187***

*CPS* Cognitive Performance Scale

*p ≤ 0.05, ***p ≤ 0.001

**Table 3:** Significant comparisons found as compared to holiday discussions (reference group)

Variable	Exercise	Games	Poetry	Baking	Choral	Storytelling	Brain games
'Engagement'		0.44; *t*_1100_ = 3.83***			0.35; *t*_1098_ = 3.18**	−0.34; *t*_1094_ = −2.92**	
Active participation	0.30; *t*_1099_ = 2.94**	0.74; *t*_1101_ = 7.29***		0.23; *t*_1099_ = 2.17*	0.35; *t*_1099_ = 3.56***	−0.22; *t*_1095_ = −2.20*	
Attitude		0.38; *t*_1113_ = 3.96***	−0.27; *t*_1109_ = −2.82**		0.45; *t*_1109_ = 4.79***	−0.27; *t*_1104_ = −2.78**	
Positive mood		0.44; *t*_1104_ = 4.26***	−0.27; *t*_1100_ = −2.63**	0.30; *t*_1101_ = 2.81**	0.38; *t*_1101_ = 3.70***		0.25; *t*_1101_ = 2.46*
Asleep		−0.29; *t*_1035_ = −2.17**			−0.31; *t*_1031_ = −2.26*		

Cells include effect size and related *t* statistic and *p* value

*p ≤ 0.05, **p ≤ 0.01, ***p ≤ 0.001

### The interaction between group topics and participants’ cognitive function

**Fig. 2:**
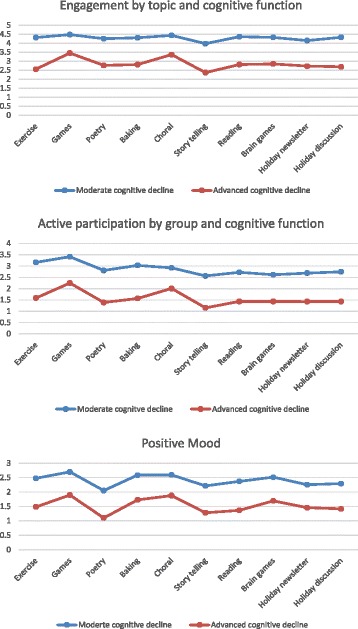
Impact of group by participants’ cognitive function

Figure 2 displays the means of ‘engagement’, active participation, and positive mood separately for participants with moderate cognitive impairment and with advanced cognitive impairment. As expected, higher levels were observed for those with moderate cognitive impairment. However, the trends were similar for both groups, suggesting that for the activities used in this study, the difference in responsiveness to the groups is a matter of magnitude of observable response rather than responsiveness to different types of groups. This is further supported by other observations: mean ‘engagement’ scores were highest for both levels of cognitive function in the games group and second highest for choral, and they were lowest for storytelling. Active participation was also highest in games for both levels of cognitive function and, again, storytelling had the lowest levels of active participation regardless of cognitive function. For positive mood, games had the highest ratings, choral had the second highest, and poetry had the lowest ratings regardless of level of cognitive functioning.

## Discussion

The results provide relatively clear answers for the three research aims. Group activities were clearly superior to control time without such groups. Participants’ levels of engagement, mood, and sleep varied with group content. While persons with higher level of cognitive function manifested more positive effects in response to the groups, the relative responses to the various group contents did not differ by cognitive function.

The superiority of group activities to control observations concords with Brooker and Duce’s [5] and Phillips et al.’s [6] findings that wellbeing was higher during activities than during unstructured time. In contrast, our findings of differential effects of groups based on their contents differ from those of some previous studies [7–9]. This could be due to our use of a larger range of activities, a larger sample size, different assessments, or the nature of the activities as described in the following. However, responses for many of the group activities in our study did not seem to significantly differ from each other, thus also providing partial agreement with those same studies.

This comparison to previous studies points to a major strength of this article, in that it uses both a large variety of activities and multiple outcome measures, which helps explain some of the negative or inconclusive findings. The results here clearly show that different activities affect different outcome measures, which supports the use of multiple outcome measures and the view of engagement as multidimensional, as specified in the GOME [2].

The most successful group activities were games and choral singing, whereas the least successful were storytelling and poetry, with other topics being in between or having a greater impact on a specific outcome, such as exercise impacting active participation. This may be partially due to the observable nature of active participation in exercise, which is likely greater than in poetry or reading. There are, however, several caveats that need to be taken into account in interpreting these findings. The group activities were conducted on the basis of a pre-prepared protocol and materials organized in a box, later labeled ‘activity in a box’ [14]. For example, for the reading activity, booklets with short stories were prepared with large print and high contrast, as well as fail-free questions for the TR staff to use with the stories; for holiday discussions, pictures, CDs, texts, and questions were prepared. The level of success of a group is a function of both the specific stimulus presented, such as songs in the choral group, as well as the particular choice of exemplars used in the activity, such as old familiar popular songs. For the less successful contents, it is possible that the particular activity was too difficult or inappropriate for the population, but it is also possible that the specifics were not sufficiently developed. For example, holiday discussion was prepared for several holidays, but, given the randomized order of the group content, groups were sometimes conducted with this topic at a calendar time that did not correspond with any of these holidays. This was a barrier to the group’s success for some of the participants, and potentially also for the TR staff. Such a barrier can potentially be addressed by preparing the activity for all holidays practiced in that part of the world. Similarly, the relative failure of the poetry activity could be related to the particular poems used rather than to the use of poetry for a discussion group.

Another issue encountered relates to ‘positive mood’ as an outcome and criterion for success. Sometimes, participants wished to discuss negative experiences. For example, one participant always brought up her experiences from the holocaust during a discussion about life events. She would usually get upset by the memory, as did some other group members, and yet she clearly wanted to discuss those experiences. Should this be counted as a negative outcome for the group activity?

This article focuses on the impact of group activities and the differences among different contents of group activities, showing that group activities are beneficial and that there are statistically significant differences among different contents of group activities in terms of their impact. Yet the results show a much greater impact of participants’ levels of cognitive functioning than of group content on the impact of groups. Indeed, many of the comparisons among groups were not statistically different. Thus, despite significant differences among the groups, the presentation of group activities may be more crucial than the specific content.

An inherent limitation to this kind of study is the use of unblinded raters. We countered this to some extent by using two types of raters: the TR staff who delivered the intervention as well as research observers who rated participants’ responses independently. Correlations between those raters were high [2], and analyses concerning the general impact of the group on engagement and mood were statistically significant for both, yet the effect sizes obtained by TR ratings were higher than those for observers (Table 1). This can be explained in two ways. First, TR staff are experienced with this population and may be better attuned to nuances in participants' reactions and behaviors. Alternately, it is possible that they are biased in wanting to see positive outcomes from their work. Both of these factors could have contributed to the results. However, there is no a priori reason to assume that staff members would be biased toward one activity or another.

Both the strengths and the limitations of the study are based on this being a clinical field study. It used one large facility in one location and its TR staff members to conduct the groups. Although TR staff ratings could be biased by their wish to see positive effects on their groups, their results were highly concordant with those of research observers [2].

## Conclusions

Group activities form an important tool in providing persons with dementia an adequate quality of life and as a nonpharmacological intervention to prevent behavior problems in this population. These benefits are based on activities diminishing boredom and loneliness in this population. This article provides the first comprehensive evidence that such groups can significantly impact engagement and mood—as compared to unstructured time. The article also shows that group content does matter, in that different contents may result in significantly different outcomes, although it appears that good use of quality groups may be more important than their content, as many group contents did not differ significantly from each other. As such, the findings provide basic building blocks for forming the science of group activities for persons with dementia. In addition, the process presented in this article of immediate assessment of the impact of group activities provides a timely feedback for detecting failures in protocols or materials or in mismatch between those who participate in the groups and the content of the activities presented. This provides information allowing for continuous improvement in the content of activities. This study demonstrated that this content makes a difference, thus indicating the importance of using such improvement mechanisms in the selection and design of activities.

## Abbreviations

CPS: Cognitive Performance Scale
df: Degrees of Freedom
MDS: Minimum Data Set
REB: Research Ethics Board
SD: Standard deviation
TR: Therapeutic recreation
